# Evaluation of maxillary sinus pneumatization amongst different age groups and genders: A CBCT based study

**DOI:** 10.6026/973206300220163

**Published:** 2026-01-31

**Authors:** Naazia Shareef, Anjali Kumari, Archana Sudheer, Lovely Kumari, Sania Heena, Meghna Mehta

**Affiliations:** 1 Department of Oral Medicine and Radiology, Buddha Institute of Dental Sciences & Hospital, Patna, Bihar, India; 2 Department of Periodontology, Buddha Institute of Dental Sciences & Hospital, Patna, Bihar, India; 3 Department of Dentistry, ESIC Medical College, Indore, Madhya Pradesh, India

**Keywords:** Maxillary sinus, alveolar process, pneumatization, cone-beam computed tomography (CBCT)

## Abstract

Excessive pneumatization can reduce the available alveolar bone height, increase the risk of surgical complications and limit prosthetic options. Therefore, it is of interest to evaluate maxillary sinus pneumatization in 200 adults across three age groups by measuring the distance between the maxillary sinus floor and nasal cavity floor at posterior tooth sites. Men showed significantly greater MSP than women in Groups I and II (p=0.004 and 0.001), while Type I pneumatization predominated across all groups, decreasing from Group I to III. Excessive MSP reduces alveolar bone height, raising surgical risks and limiting prosthetics, with variations by age, gender, and dental status aiding treatment planning.

## Background:

The maxillary sinus, located within the maxillary bone is the largest and first paranasal sinuses to develop and plays several important physiological and clinical roles. Sinus pneumatisation is a natural process which is accelerated due to tooth loss, as post extraction the bone in that area tends to undergo ridge resorption and the maxillary sinus slowly expands downward into the area where the tooth once was. As the sinus floor expands to the jaw ridge, the bone height required for advanced dental rehabilitation such as implant decreases and becomes weaker [1, 2]. The pneumatization of the maxillary sinus starts with the eruption of the permanent teeth; however, it is filled with fluid at birth. The volume expands continuously with advancing age and decreases with the attainment of puberty but continues throughout the life. This physiological process is called maxillary pneumatization (MSP) [3, 4]. The anatomical pneumatization and relations of the maxillary sinus through the alveolar bone is complex, due to the variable extension of the sinus. The relation between the teeth and the sinus floor are critical elements for diagnosis, dental treatments, and any surgical intervention of dento-antral complex [5, 6]. Due to the close anatomical proximity of the root apices of maxillary posterior teeth to the MSP, teeth infections may spread into the maxillary sinus through peri-apical tissues and cause odontogenic maxillary sinusitis. The periapical and marginal lesion of roots close to or extending into the MSF could cause inflammatory changes of sinus mucosal lining and a pathological change of maxillary sinus can also occur following improper implant therapy. All these reasons can result in various complications, such as odontogenic maxillary sinusitis, endo-antral syndrome and traumatic alterations, which are complex problems for dentists and otolaryngologists [7, 8]. MSP is known to be influenced by several factors, including age, gender, and dental status, the extent of its variation across different populations remains unclear. The introduction of CBCT has been revolutionary in the field of radio-imaging techniques. It provides a complete detailed scan of the structures evaluated, so it is used extensively in the diagnosis of orthodontics and endodontic treatments. CBCT is used to determine the density of bone, measure the alveolar bone height and width, and in reconstruction of craniofacial structures [9, 10]. With the use of Cone Beam Computed Tomography (CBCT), it is now possible to assess dimensional changes accurately in three dimensions in cases of maxillary sinus pneumatization. Therefore, it is of interest to evaluate and compare the degree of maxillary sinus pneumatization among different age groups and genders using CBCT, to better understand its clinical implications for treatment planning in dentistry.

## Materials and Methods:

## Study subjects:

This study was reviewed and approved by the Institutional Ethical Committee (approval number). The study involved the archived scans of the adult patients in the age range of 18-60. Who underwent CBCT at the Buddha Institute of dental sciences & hospital, Patna, Bihar. The data were evaluated. The study comprised of 200 scans of maxilla (102 male and 98 female) taken on ICAT CBCT machine operating at 120kvp,5mAs and exposure time of 14.7seconds with 0.25mm voxel size and field of view (FOV) of 16x6cm in maxilla.

The inclusion criteria included, Age≥18 years, No evidence of facial trauma and sinonasal surgery, No MS mucosal thickening and MS cyst or tumour, No loss of bilateral maxillary first molar and CBCT images with good quality without artefacts. The exclusion criteria for the CBCT images were as following: (1) Any evidence of trauma, fracture or pathologies (2) patients with cleft lip and palate (3) Patient with poor periodontal condition.

## CBCT images evaluations:

**Figure 1:**
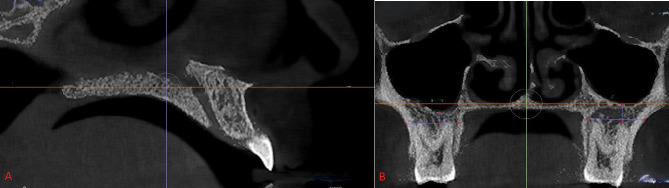
Mandibular reconstruction of the head and maxilla A Sagittal plane: the connecting line (yellow dashed line) of the anterior nasal spine was parallel to the horizontal plane; B Coronal plane: the nasal cavity floor was parallel to the horizontal plane.

**Figure 2:**
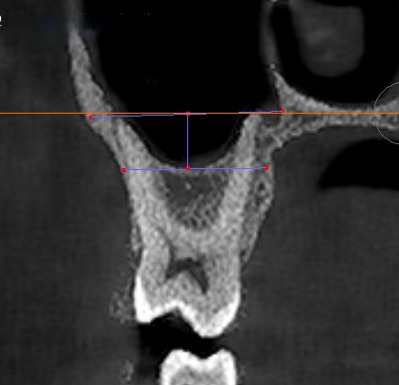
Measurement of MSP: the red line refers to the tangent of the nasal cavity floor, and the blue line arrow represents the linear distance from the nasal floor to the lowest level of the maxillary sinus floor

In the sagittal position, the connecting line of the anterior and posterior nasal spine was chosen as the palatal plane, parallel to the horizontal plane. In the coronal reconstruction, the nasal cavity floor (NCF) was positioned parallel to the horizontal plane to standardize the head and maxillary positions during image acquisition (Figure 1). The distance from the lowest level of MSF determined on a coronal plane to the NCF was defined as the amount of MSP (or MS height), based on a study by Gomes *et al.* [7] and Altayar *et al.* [9] (Figure 2). A negative value indicated that the sinus floor was above the NCF, while a positive value suggested that the sinus floor was below the NCF.

## Classification of maxillary sinus pneumatization extension into the alveolar process:

The classification proposed by Sharan *et al.* [10] and Pei *et al.* [11], was followed:

In this classification the MSP was categorized in to the following types:

1) Type I- Normal pneumatization.

2) Type II- Extensive pneumatization.

**Figure 3:**
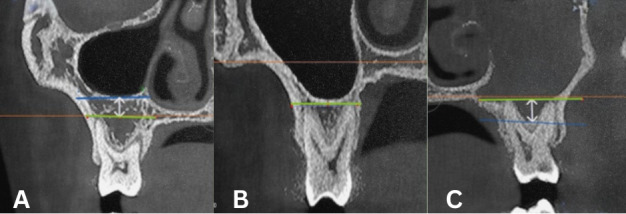
Schematic diagram of classification of maxillary sinus pneumatization. A Type I normal pneumatization; B and C Type II extensive pneumatization. Yellow dashed line: horizontal line at the sinus floor; blue dashed line: horizontal line at the root apex; white double-headed arrow: distance between the sinus floor and the root apex

In normal pneumatization, there was a certain distance between the root apex of the maxillary posterior teeth and the MSF (the MSF was apical to the level of root apex). In extensive pneumatization, the root apex of the maxillary posterior tooth was in close contact with the MSF and the root apex was located on the medial and lateral side of the MSF or protruding into the MSF (the MSF was coronal to the apex of one of the roots) (Figure 3). The maxillary posterior teeth corresponding to the teeth at the deepest position of the MSF was determined by the above method.

## Statistical analysis:

MS Excel 2016 was used to fabricate the data sheet. IBM SPSS Corp. in Armonk, New York for Windows, Version 25.0, was used for the statistical analysis. The demographic characteristics of the study population were presented in terms of frequency and percentages. Chi square statistics were applied to calculate the inferential statistics between the different variables. The statistical constant was fixed at p<0.05.

## Results:

**Figure 4:**
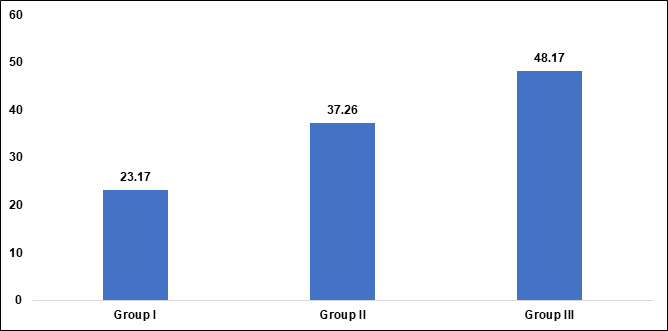
Graphical representation of age

**Figure 5:**
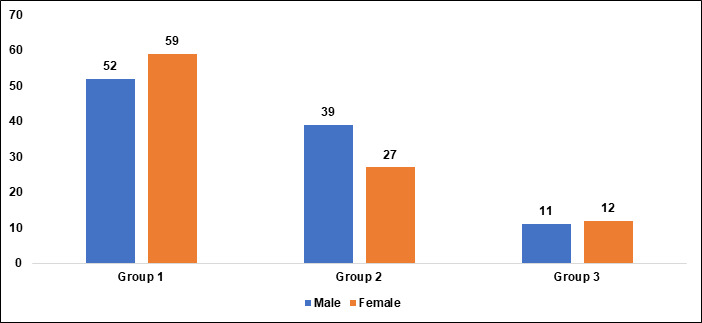
Graphical representation of gender

A total of 200 scans of maxillary sinuses were included in this study. The scans comprised of 102 males and 98 females with a mean age range of 23.17 ±3.230 years in Group I, 37.26 ±4.494 in group II and 48.17± 1.992 in Group III (Table 1, Figure 4 and Figure 5).

The table presents a comparison of three groups (Group I, Group II, and Group III) across two variables: age and gender distribution.

[1] Age - Group I have a mean age of 23.17 years with a standard deviation of 3.230, Group II has a mean age of 37.26 years with a standard deviation of 4.494 and Group III has a mean age of 48.17 years with a standard deviation of 1.992. The p-value of 0.01 indicates a statistically significant difference in age across the three groups. This suggests that as the group number increases, the average age of participants also increases significantly.

[2] Gender Distribution

1) Group I consist of 52 (46.8%) males and 59 (53.2%) females.

2) Group II has a higher percentage of males at 59.1% and a lower percentage of females at 40.9%.

3) Group III has 47.8% males and 52.2% females.

[3] The p-value of 0.001 indicates a significant difference in gender distribution among the groups, suggesting that the proportions of males and females vary significantly between the groups.

**Figure 6:**
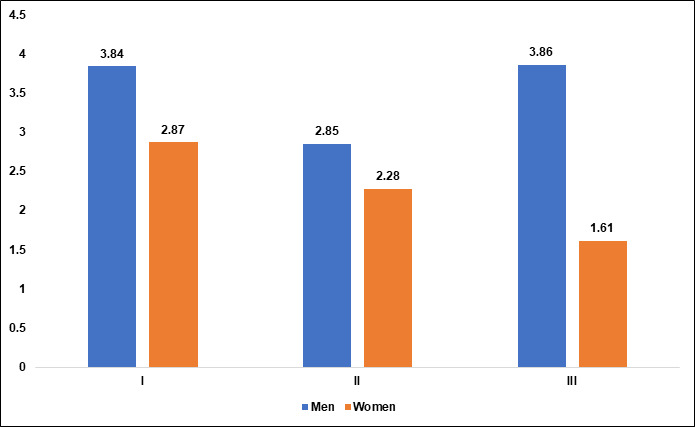
Graphical representation of the amount of maxillary sinus pneumatization extended into alveolar process based on gender of each group.

In Group I the P Value is 0.004 which indicates a statistically significant difference in the extent of sinus pneumatization based on gender, with men showing greater pneumatization than women. In group II the P Value is 0.094 which indicates that the difference in pneumatization based on gender is not statistically significant, suggesting that gender does not greatly influence maxillary sinus pneumatization in this group. In Group III the P Value is 0.001 which indicates that there is a highly significant difference, with men showing markedly greater pneumatization than women in this group (Table 2, Figure 6).

**Figure 7:**
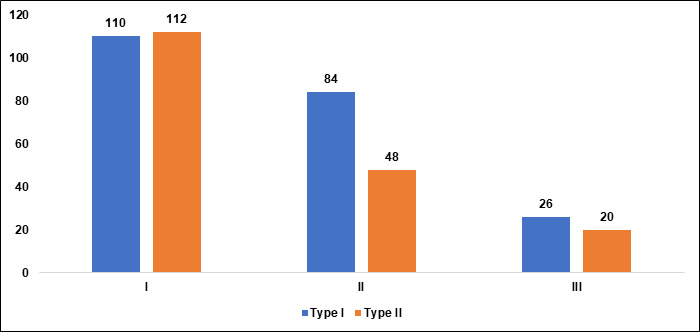
Graphical representation of classification of maxillary sinus pneumatization in different age groups

**Figure 8:**
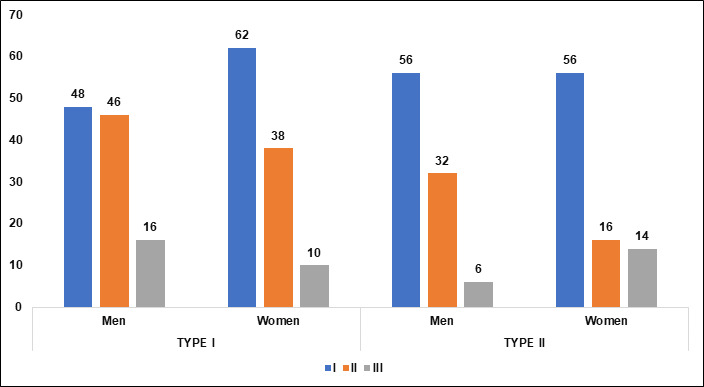
Graphical representation of types MSP among sexes and groups

In Group I, the nearly equal distribution between Type I and Type II suggests that both types are relatively common, but the statistical significance indicates meaningful differences in their prevalence. In Group II, Type I is more prevalent than Type II. The statistical significance indicates a noteworthy difference in the occurrence of the two types, with Type I being the dominant form. In Group III, there is still a higher occurrence of Type I compared to Type II, although the overall numbers are smaller. The significant p-value reinforces the presence of a notable difference, though both types are less common in this group compared to the previous ones. Overall, the analysis shows that Type I pneumatization is more prevalent across all age groups, but its relative prevalence decreases from Group I to Group III. As age increases, the overall number of participant's decreases, but Type I remains the more common type, suggesting that certain anatomical or developmental factors associated with aging may influence the degree of pneumatization (Table 3, Figure 7). In Group I, there are more women than men in Type I pneumatization, and the distribution for Type II is equal between genders. The significant p-value suggests that gender has an impact on the distribution of pneumatization types within this group. In Group II, there are more men than women in both Type I and Type II pneumatization. The significant p-value indicates a meaningful difference in the distribution of the types between genders, with men showing a higher prevalence of both types. In Group III, men again have a higher count for Type I, while women have a higher count for Type II. The significant p-value points to a noteworthy difference in how pneumatization types are distributed between men and women in this group as well (Table 4, Figure 8).

## Discussion:

The study evaluated the amount and degree of MSP with age, gender, and MSP in to the alveolar process among different groups and gender in a cohort of 200 patients. The patient was divided into three age groups with Group I having a mean age of 23.17 years with a standard deviation of 3.230, Group II has a mean age of 37.26 years with a standard deviation of 4.494 and Group III has a mean age of 48.17 years with a standard deviation of 1.992 respectively. The p-value of 0.01 indicates a statistically significant difference in age across the three groups. This suggests that as the group number increases, the average age of participants also increases significantly. When the amount of maxillary sinus pneumatization extending into alveolar process of maxilla based on gender of each group was compared, it was found that group I and group II is statistically significant with P value of 0.004 and 0.001 with men showing greater pneumatization than women in both the groups. Alqahtani H *et al.* in their study found that the occurrence of MSP increases significantly after extraction of tooth thus reducing the height of the bone needed for placement of implants [12]. Similarly, when the type of maxillary sinus pneumatization was compared among different age groups, it was found that Type I pneumatization is more prevalent across all age groups, but its relative prevalence decreases from Group I to Group III. As age increases, the overall number of participants decreases, but Type I remains the more common type, suggesting that certain anatomical or developmental factors associated with aging may influence the degree of pneumatization. The process of Pneumatization starts from birth and progresses throughout life and attaining stability in the adulthood phase, the amount of pneumatization is higher in the younger age group due to various factors such as height and body mass index , dietary habits like consumption of more processed foods which needs less occlusal forces and chewing and less functional stimulation during development of jaw as compared to older age groups thereby leading to significant increase in MSP in younger groups [2]. The pneumatization of maxillary sinus when compared among both genders, it was found that there is a statistically significant difference in the distribution of maxillary sinus pneumatization types between genders across all three groups. In Group I, women show a higher prevalence of Type I, while men are equally distributed in Type II. In Group II, men show a clear dominance in both types and in Group III, men have a higher prevalence of Type I, but women outnumber men in Type II. Few studies have reported [13, 14] reported a statistically significant difference in the amount of MSP between males and females, with males showing greater MS volume than females. The increased prevalence in males maybe attributed to their larger skull proportion and body mass [15, 16, 17].

## Conclusion:

The amount of MSP was significantly associated with patients' age, showed a decreasing trend with increasing age and was more prevalent in the younger years old group, while it was not significantly associated with gender. Therefore, considering the consequences of MSP extended into the alveolar process, it could be important to determine the amount and degree of MSP before treatment of the maxillary posterior region, especially in high-risk patients, such as those aged 18-25 years old.

## Figures and Tables

**Table 1:** Age and gender characteristics of the three groups

**Variables**	**Group I (n=111)**	**Group II (66)**	**Group III(n=23)**	**P value**
Age in years	23.17 ± 3.23	37.26 ± 4.494	48.17 ± 1.992	0.01*
Male	52(46.8%)	39(59.1%)	11(47.8%)	0.001*
Female	59(53.2%)	27(40.9%)	12(52.2%)	0.001*

**Table 2:** Comparison of the amount of maxillary sinus pneumatization extending into alveolar process of maxilla based on gender of each group.

**GROUP**	**n**	**Mean ± SD (mm)**	**Gender**		**F Score**	**P value**
			**Men**	**women**		
I	222	3.34±2.48	3.84±2.88 (n=104)	2.87±2.14 (n=118)	8.273	.004*
II	132	2.56±1.86	2.85±2.07 (n=78)	2.28±1.65 (n=54)	2.84	0.094
III	46	2.73±2	3.86±2.64 (n=22)	1.61±1.36 (n=24)	13.45	.001*

**Table 3:** Classification of maxillary sinus pneumatization (MSP) in different age groups

**Group**	**Type I(n=220)**	**Type II(n=180)**	**P value**	**Prevalence %**
I	110	112	0.001*	55.5
II	84	48	0.01*	33
III	26	20	0.01*	11.5
*Statistically significant

**Table 4:** Classification of maxillary sinus pneumatization (MSP) among both genders

**GROUP**	**TYPE I (n=110)**		**TYPE II (n=112)**		**P value**
	**Men**	**women**	**Men**	**women**	
I	48	62	56	56	0.01*
II	46	38	32	16	0.01*
III	16	10	6	14	0.01*
